# The Impact of High-Fat Diet and Restrictive Feeding on Natural Killer Cells in Obese-Resistant BALB/c Mice

**DOI:** 10.3389/fnut.2021.711824

**Published:** 2021-07-23

**Authors:** Julia Spielmann, Wiebke Naujoks, Matthias Emde, Martin Allweyer, Johannes Fänder, Heike Kielstein, Dagmar Quandt, Ina Bähr

**Affiliations:** Institute of Anatomy and Cell Biology, Medical Faculty of Martin Luther University Halle-Wittenberg, Halle, Germany

**Keywords:** NK cells, overweight, obesity, cytotoxicity, NK cell receptors, NK cell subsets, cancer, BALB/c

## Abstract

**Background:** The association of obesity and an increased risk for severe infections and various cancer types is well-described. Natural killer (NK) cells are circulating lymphoid cells and promoters of the immune response toward viruses and malignant cells. As demonstrated in previous studies the phenotype and functionality of NK cells is impaired in obesity. So far, the majority of animal studies were exclusively performed using *ad libitum* feeding regimes and it remained unclear whether NK cell alterations are mediated by obesity-associated immunological changes or by direct effects of the dietary composition. Therefore, the aim of the present study was to characterize NK cells in the peripheral blood of obese-resistant BALB/c mice supplied a normal-fat diet (NFD) or high-fat diet (HFD), *ad libitum* or in a restrictive manner.

**Methods:** Twenty-eight BALB/c-mice were fed a NFD or HFD either *ad libitum* or in a restrictive feeding regime with 90% of the mean daily diet supply of the corresponding *ad libitum* group (each group *n* = 7). Blood and visceral adipose tissue were collected for flow cytometric analysis, analysis of plasma cytokine concentrations by multiplex immunoassay and real-time RT-PCR analyses. For statistical analyses two-way ANOVA with the factors “feeding regime” and “diet” was performed followed by a *post-hoc* Tukey's multiple comparison test and to compare means of the four mouse groups.

**Results:** *Ad libitum*-feeding of a HFD in BALB/c mice has no influence on body weight gain, visceral fat mass, plasma cytokine concentrations, immune cell populations as well as the number, frequency and phenotype of NK cells. In contrast, restrictive feeding of a HFD compared to NFD led to significantly higher body weights, visceral fat mass and plasma interferon-γ concentrations which was associated with changes in the frequencies of granulocytes and NK cell subsets as well as in the surface expression of NK cell maturation markers.

**Conclusion:** Results demonstrate for the first time that HFD-induced alterations in NK cells are consequences of the obese associated immunological profile rather than a direct effect of the dietary composition. These data can help to clarify the increased risk for cancer and severe infections in obesity.

## Introduction

Overweight and obesity are major public health challenges of this century. The prevalence for overweight and obesity is persistently rising and reaches pandemic levels. According to the World Health Organization (WHO), more than 1.9 billion adults were overweight and, of these, over 650 million adults were obese in 2016 (1). This alarming situation is prevalent in developing as well as in developed countries and affects all ages and socioeconomic groups. Excess accumulation of body fat is associated with an increased risk for numerous disorders, like coronary heart disease or diabetes, and an increased risk for various cancer types, such as postmenopausal breast, colorectal or kidney cancer (2–5). Moreover, recent studies clearly demonstrated that obese individuals have a higher susceptibility to infections as well as a more severe progression of the infectious disease, like infections with influenza A or SARS-CoV-2 (6, 7). Moreover, obesity is associated with an increased overall mortality rate (1, 8). In addition to numerous changes of metabolic parameters, obese condition leads to a subclinical systemic low-grade inflammation as well as functional alterations of various immune cells, like macrophages or natural killer (NK) cells (9–11).

NK cells are innate lymphoid cells and play a crucial role in host defense of cancer and virus-infected cells. NK cells in humans are specified as CD3 negative lymphocytes expressing CD56. Two major subpopulations of human NK cells are usually defined based on their surface expression of CD56 and CD16—the more immunoregulatory CD56^bright^ NK cell subset and the CD56^dim^ NK cell subset with greater cytotoxic capacities (12). Similar, in mice, NK cells are identified as CD3 negative cells expressing NKp46 (CD335), NK1.1 (CD161) or CD49b (DX5). Moreover, based on the differential expression of CD11b and CD27, murine NK cell subpopulations have been characterized according to the 4-stage model of NK cell maturation—CD11b^low^CD27^low^, CD11b^low^CD27^high^, CD11b^high^CD27^high^, and CD11b^high^CD27^low^ NK cells (13, 14). As the developmental progression is associated with enhanced NK cell functions, cytolytic activity and cytokine secretion is the highest in the CD11b^high^CD27^high^ NK cell subset (13, 15).

NK cells eliminate target cells by engaging death receptors that initiate caspase cascades or by releasing cytotoxic granules enclosing perforin and granzymes. Moreover, NK cells mediate immunoregulatory functions by secretion of several cytokines, like interferon (IFN)-γ or tumor necrosis factor (TNF)-α (12). The activity of NK cells is controlled by a balanced expression of inhibitory, costimulatory, and activating receptor (16). Additionally, the expression of NK cell receptor ligands on target cells is mandatory for activating the cytolysis and cytokine production of NK cells.

In mice, most pivotal activating NK cell receptors are the natural cytotoxicity receptor NKp46, the natural killer group (NKG) 2D receptor and the activating members of the lectin-like Ly49 receptor family—the murine analog for human killer immunoglobulin-like receptors (KIRs). Inhibitory NK cell receptors include the killer cell lectin-like receptor (KLR) subfamily G1, the NKG2A receptor and the inhibitory Ly49 receptors (17).

Previous studies on rodents and humans demonstrated that the phenotype, number and functionality of NK cells are impaired in obesity (10, 18). Most of the animal studies investigating NK cell functionality in genetically or diet-induced obesity in mice were performed in (i) male, (ii) C57BL/6 mice or (iii) in mutated C57BL/6 mice lacking leptin production (ob/ob mice) or processing deficient leptin receptors (db/db mice) (10). However, some previous investigations demonstrated that the use of different mouse strains or sex is not only associated with large variances in body weight, fat mass or metabolic factors, but also in immune cell function considering different mouse models used in the studies (19–22). Moreover, in studies investigating the impact of excess body weight on NK cells, obesity was exclusively induced under an *ad libitum* feeding regime of a high-fat diet. It is well-known that the development of obesity including metabolic and immunological parameters can be affected by various feeding regimes, like time-or energy-restricted feeding (23–26). We recently published a study on C57BL/6 mice and could show that obesity-related alterations on immune cells including NK cells can be partially prevented by feeding the HFD restrictively in contrast to *ad libitum* feeding (18). Until now it is unclear whether these NK cell alterations are caused by effects of the high-fat diet itself or by metabolic alterations, like low-grade inflammation or adipokines secretion, induced by the excess body weight in obesity. Therefore, the purpose of thisstudy was to characterize NK cells in peripheral blood of female obesity-resistant BALB/c mice fed a control or high-fat diet using *ad libitum* or restrictive feeding regimes.

## Materials and Methods

### Animal Husbandry, Dietary Regimes, and Experimental Setup

Animal experiments on BALB/c mice were performed as previously published for studies on C57BL/6 mice (18). In brief, 6 weeks old female BALB/c mice (*N* = 28) were exposed to a light regime of 12-h light and 12-h dark, a constant temperature of 23 ± 2°C and 55 ± 5% relative humidity with water accessible *ad libitum*. After 1 week of adapation under *ad libitum* feeding condition with common rodent chow (Altromin, Lage, Germany), mice were housed individually in four randomized groups. In detail, BALB/c mice were fed either a normal-fat diet (NFD with 10 %kcal fat; D12450J, Research Diets, New Brunswick, USA; *n* = 14), or a high-fat diet (HFD with 60 %kcal fat; D12492– matches the sucrose calories in D12450J, Research Diets; *n* = 14) for 17 weeks. The diet composition is demonstrated in Supplementary Table 1. Moreover, mice were fed the NFD or HFD either *ad libitum* (*n* = 7 for NFD; *n* = 7 for HFD) or in a restrictive manner with 90% of the mean daily diet supply of the according *ad libitum* group (*n* = 7 for NFD; *n* = 7 for HFD). Mice fed the NFD or HFD restrictively received the diet daily at the beginning of the dark active phase. Food consumption was recorded daily and the according food amount for the restrictive fed groups was determined based on the daily food intake of the respective *ad libitum* group of the day before. The daily energy intake and the intake of nutritional components was determined using the daily food consumption and information on diet composition provided by the manufacturer. Mouse body weights were recorded weekly throughout the whole experimental period. Final body weights were determined 17 weeks after starting the dietary intervention. All research and animal care protocols were approved by the local committee for animal care (reference number 42502–2-1341 MLU) and the principles of laboratory animal care according to the guidelines of the Federation of Laboratory Animal Science Associations (FELASA) and the German Society for Laboratory Animal Science (GV-SOLAS) were followed.

### Mouse Anesthesia, Sacrificing, and Probe Sampling

At the end of the feeding period, mice were sacrificed under isoflurane inhalation anesthesia (1.5–2.0% v/v in O_2_) and blood was collected by cardiac puncture into EDTA-coated tubes. Approximately 500 μl of blood were used for subsequent flow cytometric analysis. For cytokine measurements, plasma was obtained by a centrifugation step and stored at −80°C until analyzed. Visceral adipose tissue was removed, and the weight was determined. Samples of adipose tissue samples were frozen at once in liquid nitrogen and stored at −80°C until analyzed.

### Analyses of Plasma Cytokines

In line with previously published procedures, plasma cytokine concentrations were analyzed using a multiplex immunoassay (High Sensitivity 5-Plex Mouse ProcartaPlex™ Panel; Thermo Fisher Scientific, Darmstadt, Germany) following the manufacturer's recommendations (18). Concentrations of plasma cytokines were quantified using the LiquiChip luminex 200 system (Qiagen, Hilden, Germany) and the Procartaplex-analyst 1.0 software (Affymetrix, eBioscience, San Diego, USA).

### Flow Cytometric Analyses of Immune Cells in Peripheral Blood

Multicolor flow cytometry analyses were performed as previously described (18). In brief, to analyze different leucocyte subsets and the surface expression of NK cell receptors by flow cytometry, the whole blood of mice was stained with an appropriate combination of monoclonal fluorochrome-conjugated antibodies for 15 min at RT protected from light. Antibodies used for surface staining were presented in Supplementary Table 2. To obtain leukocytes, red blood cell (RBC) lysis buffer (c.c. pro GmbH, Oberdorla, Germany) was added and samples were incubated for 10 min in order to eliminate RBCs. Afterwards, samples were rinsed twice with FACS buffer containing phosphate buffered saline (PBS; Merck), 1% fetal bovine serum (Merck KGaA, Darmstadt, Germany), 2 mM EDTA and 0.05% sodium azide (both Carl Roth, Karlsruhe, Germany), resuspended in FACS buffer and stored at 4°C protected from light for further analysis.

To determine specific surface molecules on NK cells and NK cell subpopulations, a backbone staining with the antibodies CD45, CD3, and CD335 was included in every single panel tube in order to differentiate immune cell populations. In addition, one tube containing only these antibodies served as a Fluorescence Minus One (FMO) control. Moreover, Trucount tubes (Trucount™ Tubes, BD Biosciences, Sa Jose, USA) with a defined number of fluorescent beads were used to determine absolute leukocyte numbers in the blood. Data were further processed using FlowJo version 8.7 (FlowJo LLC, Ashland, USA) andBD FACS Diva™ software version 7.0 (Becton Dickinson). For the immunophenotyping of NK cells, 50,000–100,000 events were analyzed per panel. The gating strategies identifying immune cell populations, NK cell subpopulations and NK cell phenotyping of surface markers is demonstrated in Supplementary Figure 1. Whenever gating for positive cells was possible, percentages are given, otherwise median fluorescence intensity for individual markers are presented.

### Real-Time RT-PCR Analyses

As previously published, total RNA of frozen adipose tissue was isolated by means of the RNeasy® Lipid Tissue Mini Kit (Qiagen, Hilden, Germany) according to the supplier's recommendations (18). Subsequently, reverse transcription into cDNA was performed using the ThermoScript RT-PCR kit (Thermo Fisher Scientific Inc., Darmstadt, Germany) in compliance with the manufacturer's instructions. Quantitative real-time PCR was conducted using the SYBR Green Fluorescein Mix (BioRad, München, Germany) and the qTOWER3 thermocycler (Analytik Jena AG, Jena, Deutschland). Using the ΔΔCt method and peptidylprolyl isomerase A (PPIA) as housekeeping gene, relative expression levels of samples were determined. Details of primer pairs are presented in Supplementary Table 3. Efficiency of amplification for each primer pair was generated from the slope of the standard curve using various primer dilutions.

### Statistical Analyses

All statistical analyses were conducted using the GraphPad Prism software version 7.03 (La Jolla, USA). Identification of outliers was performed using GraphPad Prism's robust regression and outlier removal (ROUT) method. Subsequently, tests fornormal distribution and homogeneity of variances were conducted. To compare means of the four mouse groups, two-way ANOVA with the main factors “feeding regime” and “diet” following Tukey's multiple comparison *post-hoc* test. Results are indicated as means ± standard error of the mean (SEM). Significance level was set at *p* ≤ 0.05. According to results of the *post-hoc* Tukey's multiple comparison test, means with different letters (a, b, c, d) denote significant differences between mice groups.

## Results

### Dietary Intake

Feeding a high-fat diet as well as restrictive feeding had significant effects on food and energy intake as well as carbohydrate, protein and fat consumption (Supplementary Table 4).

Mice fed the HFD *ad libitum* or restrictively had a significantly decreased daily intake of food and carbohydrates and a significant increased daily intake of fat. Moreover, the daily energy and protein intake was significantly lower in HFD-fed mice compared to NFD-fed mice unaffected by the feeding regime (Supplementary Table 4). Feeding in a restrictive manner resulted in a significantly lower daily intake of food amount, energy, proteins and carbohydrates in both NFD and HFD-fed mice. In contrast, the daily fat intake was decreased in mice fed the HFD restrictively compared to mice fed the HFD *ad libitum*, whereas no influence of the feeding regime was observed in NFD-fed mice (Supplementary Table 4).

### Body Weight and Visceral Fat Mass

**Figure 1:**
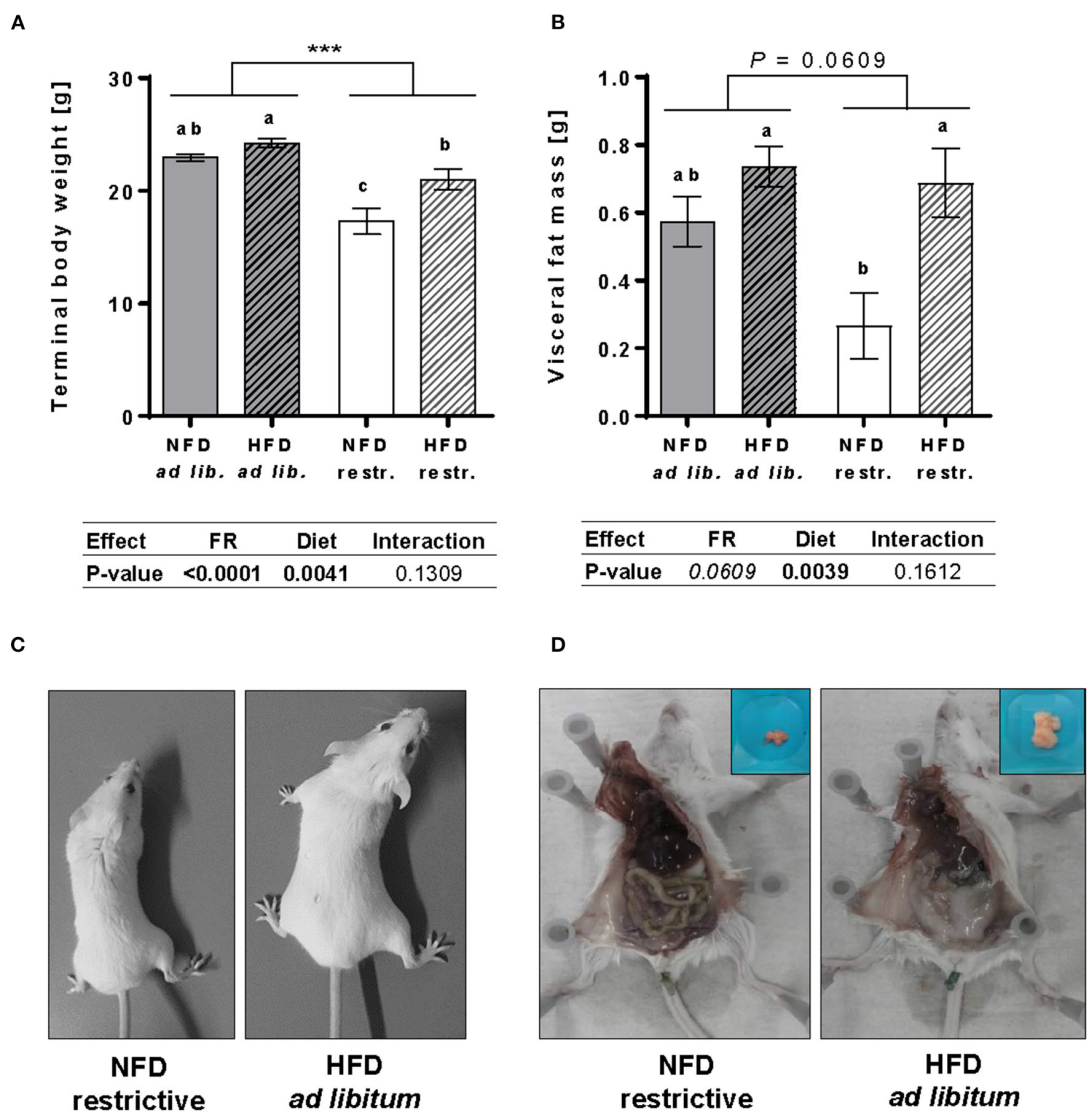
Impact of feeding a high-fat diet (HFD) and normal-fat diet (NFD) under *ad libitum* (ad lib.) and restrictive (restr.) feeding schemes in female BALB/c mice. Final body weight **(A)** and visceral fat mass **(B)** after 18 weeks of feeding the diets. Typical pictures showing visual differences in body weight **(C)** and visceral fat mass **(D)** of BALB/c mice fed NFD restrictive vs. HFD *ad libitum*. Data are presented as mean ± SEM. Different letters indicate means with significant differences according to *post-hoc* Tukey's multiple comparison test (*P* ≤ 0.05). Data tables indicate *P*-values of two-way ANOVA analyses with the main factors “feeding regime (FR)” and “diet”. Significant differences are printed in bold type. Italic *P*-values indicate a tendency to significance (0.5 ≤ *P* ≤ 0.1). ^***^P ≤ 0.001, two-way ANOVA, *ad libitum*-fed groups compared to restrictive-fed groups; exact *P*-values within 0.05 ≤ *P* ≤ 0.1 are depicted.

Results demonstrated that feeding a HFD resulted in significantly higher body weights and visceral fat mass compared to NFD-fed mice unaffected by the feeding regime. Single-group analyses showed that feeding a HFD led to significantly higher terminal body weights and visceral fat mass in mice fed restrictively, whereas no significant differences in terminal body weights or visceral fat mass were detected in mice fed the NFD or HFD *ad libitum* (Figures 1A,B). Restrictive feeding of NFD or HFD led to significantly lower terminal body weights compared to the corresponding *ad libitum* groups (Figure 1A). In contrast, restrictive feeding had no significant effect on the visceral fat mass of NFD- and HFD-fed mice (Figure 1B). In Figures 1C,D, representative pictures show visual differences in body weights and visceral fat mass of BALB/c mice fed the NFD restrictively and BALB/c mice fed the HFD *ad libitum*.

### Plasma Cytokine Concentrations

**Figure 2:**
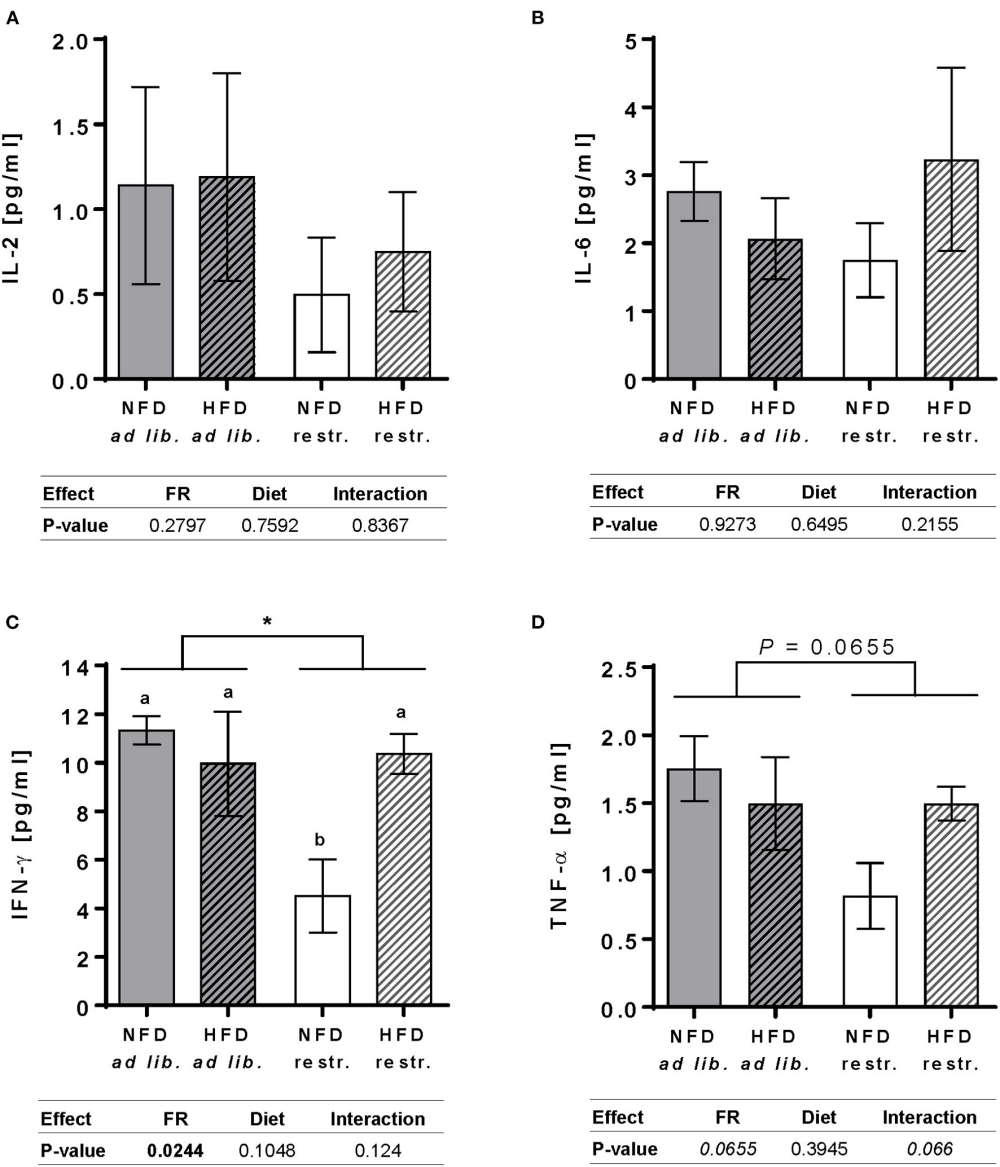
Plasma cytokine levels of interleukin (IL)-2 **(A)**, IL-6 **(B)**, interferon (IFN)-γ **(C)** and tumor necrosis factor (TNF)-α **(D)** in BALB/c mice fed a high-fat diet (HFD) or normal-fat diet (NFD) under *ad libitum* (ad lib.) and restrictive (restr.) feeding schemes quantified by multiplex analyses. Data are presented as mean ± SEM. Different letters indicate means with significant differences according to *post-hoc* Tukey's multiple comparison test results (*P* ≤ 0.05). Data tables indicate *P*-values of two-way ANOVA analyses with the main factors “feeding regime (FR)” and “diet”. Significant differences are printed in bold type. Italic *P*-values indicate a tendency to significance (0.5 ≤ *P* ≤ 0.1). ^*^P ≤ 0.05, two-way ANOVA, *ad libitum*-fed groups compared to restrictive-fed groups.

In restrictive-fed mice, feeding a HFD compared to NFD led to significantly increased plasma concentrations of IFN-γ, but not in *ad libitum*-fed mice (Figure 2C). Moreover, restrictive feeding led to significantly reduced IFN-γ plasma concentrations in NFD-fed animals, but not in HFD-fed animals, compared to the respective *ad libitum*-fed groups (Figure 2C). No significant differences between all groups were revealed for IL-2, IL-6, and TNF-α plasma concentrations (Figures 2A,B,D).

### Analyses of Immune Cell Populations in Peripheral Blood

**Figure 3:**
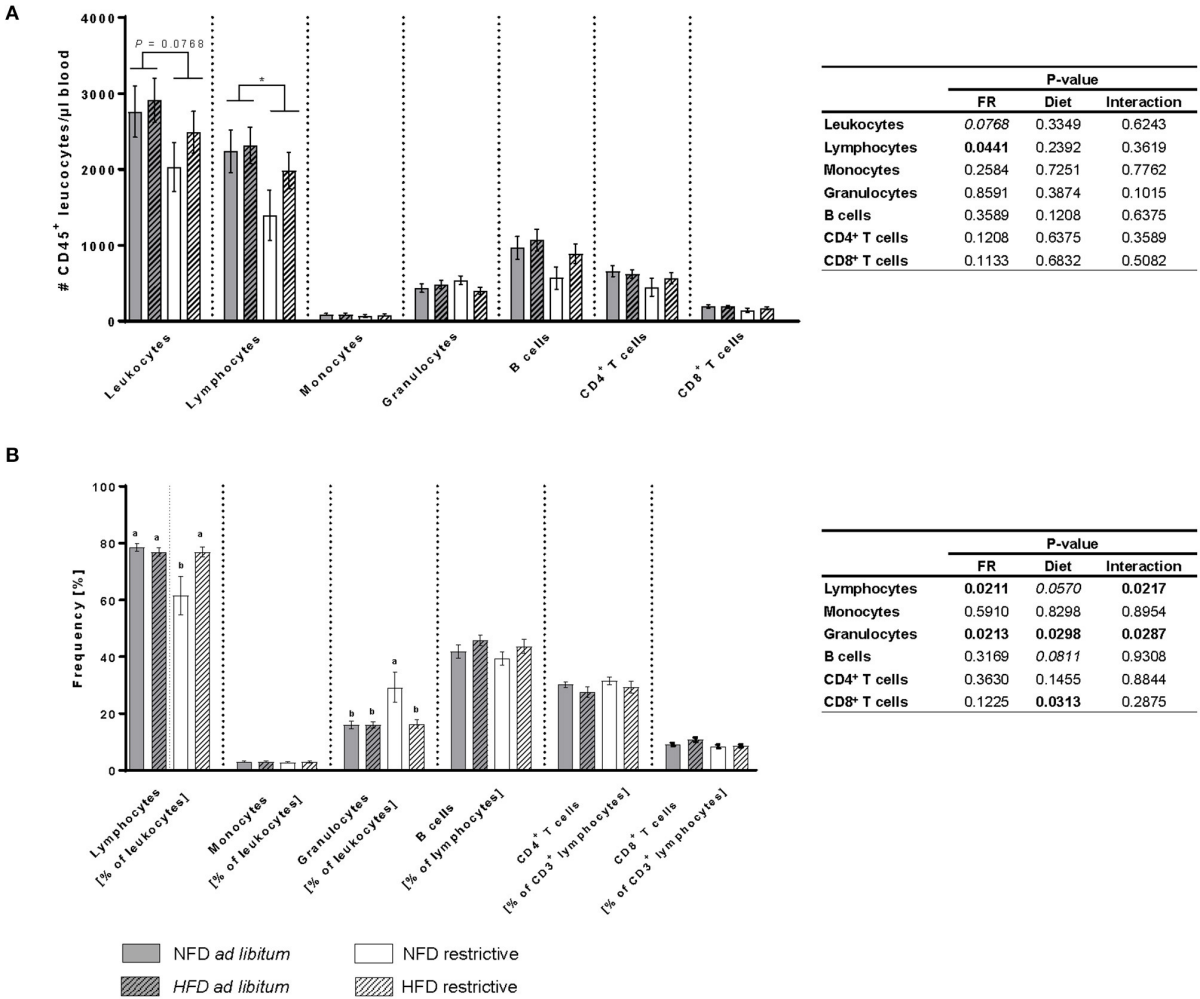
Flow cytometric analyses of absolute cell counts **(A)** and frequencies **(B)** of immune cell populations in blood samples of BALB/c mice fed a high-fat diet (HFD) or normal-fat diet (NFD) under *ad libitum* (ad lib.) and restrictive (restr.) feeding schemes. Data are presented as mean ± SEM. Different letters indicate means with significant differences according to *post-hoc* Tukey's multiple comparison test results (*P* ≤ 0.05). Data tables indicate *P*-values of two-way ANOVA analyses with the main factors “feeding regime (FR)” and “diet.” Significant differences are printed in bold type. Italic *P*-values indicate a tendency to significance (0.5 ≤ *P* ≤ 0.1). *P 0.05, two-way ANOVA, *ad libitum*-fed groups compared to restrictive-fed groups.

The total cell count of lymphocytes was significantly decreased in restrictively-fed mice compared to *ad libitum*-fed mice unaffected by the feeding regime (Figure 3A). Additionally, the frequency of lymphocytes was significantly decreased in mice fed the NFD restrictively compared to the three other mice groups (Figure 3B). In contrast, the frequency of granulocytes was significantly higher in mice fed the NFD restrictively compared to the three other experimental groups (Figure 3B). Moreover, analyses showed significantly lower frequencies of CD8^+^ T cells in HFD-fed mice compared to NFD-fed mice independent of the feeding regime (Figure 3B). No significant differences were observed in the total cell counts of leucocytes, monocytes, granulocytes, B cells as well as CD4^+^ and CD8^+^ T cells and the frequencies of monocytes, B cells and CD4^+^ T cells (Figures 3A,B).

### Investigations on Total NK Cells and NK Cell Subpopulations in Peripheral Blood

**Figure 4:**
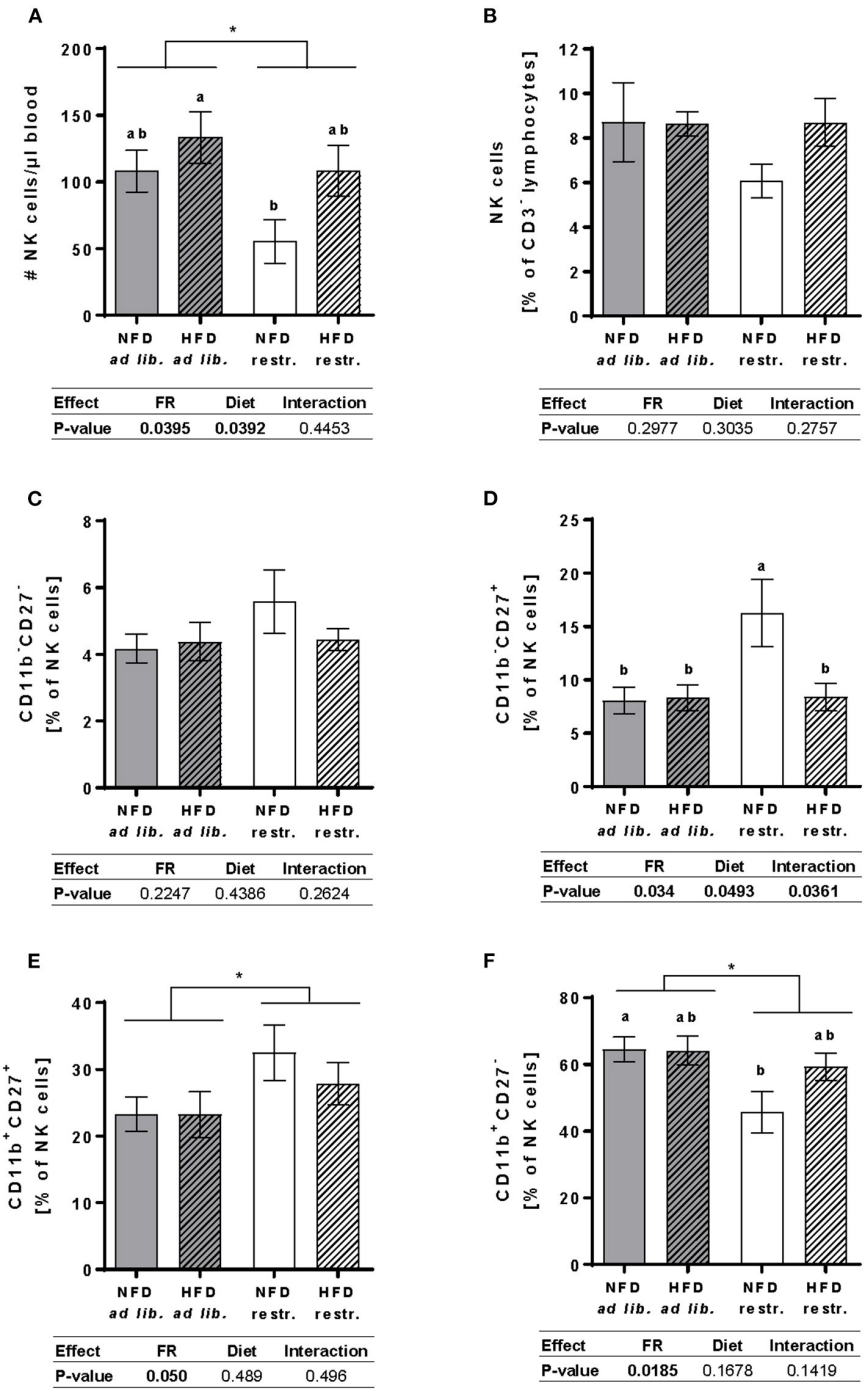
Multicolor flow cytometric analyses of total NK cells and NK cell subpopulations in blood samples of BALB/c mice fed a high-fat diet (HFD) or normal-fat diet (NFD) under *ad libitum* (ad lib.) and restrictive (restr.) feeding schemes. Concentration of total NK cells **(A)**, given as absolute cell number per μl, and percentages of total NK cells **(B)**. Percentages of NK cell subpopulations with distinct surface expression profiles of CD11b and CD27 **(C–F)**. Data are presented as mean ± SEM. Different letters indicate means with significant differences according to post-hoc Tukey's multiple comparison test results (*P* ≤ 0.05). Data tables indicate *P*-values of two-way ANOVA analyses with the main factors “feeding regime (FR)” and “diet.” Significant differences are printed in bold type. *P ≤ 0.05, two-way ANOVA, *ad libitum-*fed groups compared to restrictive-fed groups.

Results demonstrated that the total cell counts of circulating NK cells was significantly increased in mice fed the HFD compared to mice fed the NFD unaffected by the feeding regime. Moreover, restrictive feeding resulted in significantly reduced NK cell concentrations compared to *ad libitum*-fed mice independent of the diet. Single-group analyses demonstrated significantly decreased NK cell concentrations in mice fed the NFD restrictively compared to mice fed the HFD *ad libitum* (Figure 4A). In contrast, no significant differences in the NK cell frequency was detected between the four mice groups (Figure 4B).

Investigations on NK cells subsets demonstrate that the diet and feeding regime did not significantly affect the frequency of the CD11b^−^CD27^−^ NK cell subset (Figure 4C). The frequency of the CD11b^−^CD27^+^ NK cell subset was significantly higher in mice fed the NFD restrictively compared to the three other experimental groups (Figure 4D). Moreover, restrictive feeding resulted in an increased frequency of the CD11b^+^CD27^+^ NK cell subset unaffected by the feeding regime (Figure 4E). In contrast, results demonstrated that restricted feeding reduced the frequencies of CD11b^+^CD27^−^ NK cells independent of the diet. Single-group analyses revealed a significant decrease of the CD11b^+^CD27^−^ NK cell subpopulation in mice fed the NFD restrictively compared to mice fed the NFD *ad libitum* (Figure 4F).

### Analyses of the Surface Receptor Expression of Activating and Inhibiting NK Cell Receptors in Peripheral Total NK Cells and NK Cell Subpopulations

In addition to investigations on total NK cells and NK cell subpopulations, the impact of different feeding regimes and HFD on the expression of activating and inhibitory NK cell receptors as well as maturation markers and adhesion molecules was analyzed in the peripheral blood of BALB/c mice.

**Figure 5:**
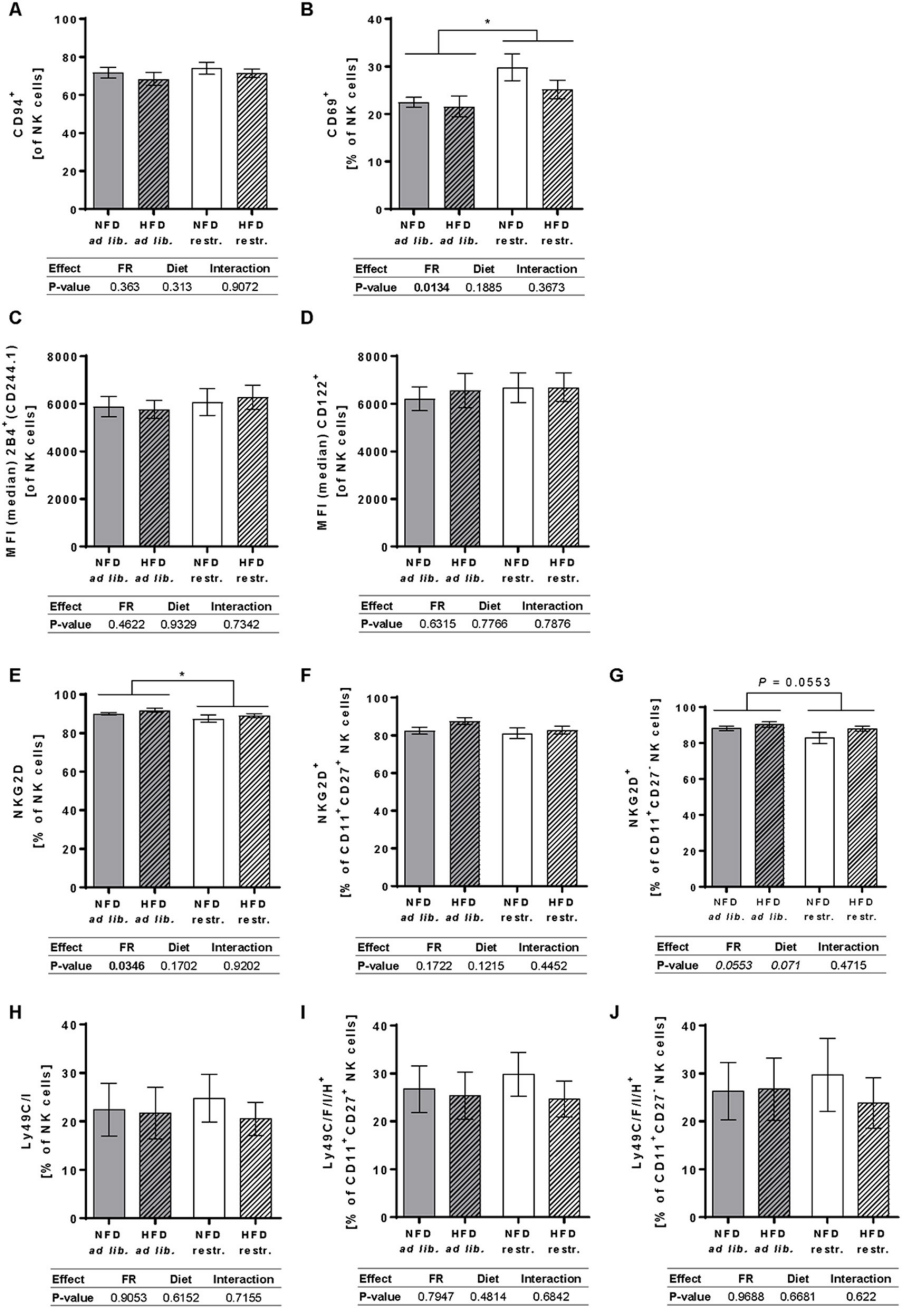
**(A–C)** Multicolor flow cytometric analyses of frequencies or median fluorescent intensities (MFIs) of total NK cells of BALB/c mice expressing the activating receptor CD94 **(A)**, the activation-associated receptor CD69 **(B)** or the co-activating receptors 2B4 **(C)** and CD122 **(D)**. **(E–J)** Frequencies of total NK cells, CD11b^+^CD27^+^NK cells and CD11b^+^CD27^−^ NK cells of BALB/c mice expressing the NKG2D, an activating receptor **(E–G)**, or the inhibitory and activating receptors Ly49C/F/I/H **(H–J)**. Mice were fed a high-fat diet (HFD) or normal-fat diet (NFD) under *ad libitum* (ad lib.) or restrictive (restr.) feeding schemes. Data are presented as mean ± SEM. Different letters indicate means with significant differences according to *post-hoc* Tukey's multiple comparison test results (*P* ≤ 0.05). Data tables indicate *P*-values of two-way ANOVA analyses with the main factors “feeding regime (FR)” and “diet.” Significant differences are printed in bold type. Italic *P*-values indicate a tendency to significance (0.5 ≤ *P* ≤ 0.1). *P ≤ 0.05, two-way ANOVA, *ad libitum*-fed groups compared to restrictive-fed groups; exact *P*-values within 0.05 ≤ *P* ≤ 0.1 are depicted.

Results demonstrate a significant increase of the frequency of total NK cells expressing the activation-associated receptor CD69 in restrictive-fed mice in comparison to *ad libitum*-fed mice unaffected by the diet (Figure 5B). Moreover, restrictive feeding resulted in a decreased frequency of total NK cells expressing NKG2D, an activating receptor, compared to the *ad libitum* feeding regime independent of the diet, whereas no significant differences were observed in the frequency of NKG2D^+^ cells in the CD11b^+^CD27^+^ and CD11b^+^CD27^−^ NK cell subsets (Figures 5E–G). In addition, no significant effects on the frequencies of total NK cells expressing the activating receptor CD94 as well as in the MFIs of the co-activating receptors 2B4 and CD122 in total NK cells were detected comparing all mice groups (Figures 5A,C,D).

The staining of Ly49 receptors was performed using anantibody that recognizes a common epitope of the activating receptor Ly49H and the inhibitory receptors Ly49C, F and I, but not other Ly49 members. Analyses of the Ly49C/F/I/H surface expression on total NK cells as well as the CD11b^+^CD27^+^ and CD11b^+^CD27^−^ NK cell subpopulation showed no significant differences between the four experimental groups (Figures 5H–J).

### Analyses of the Surface Receptor Expression of Adhesion Molecules and Maturation Markers in Peripheral Total NK Cells and NK Cell Subpopulations

**Figure 6:**
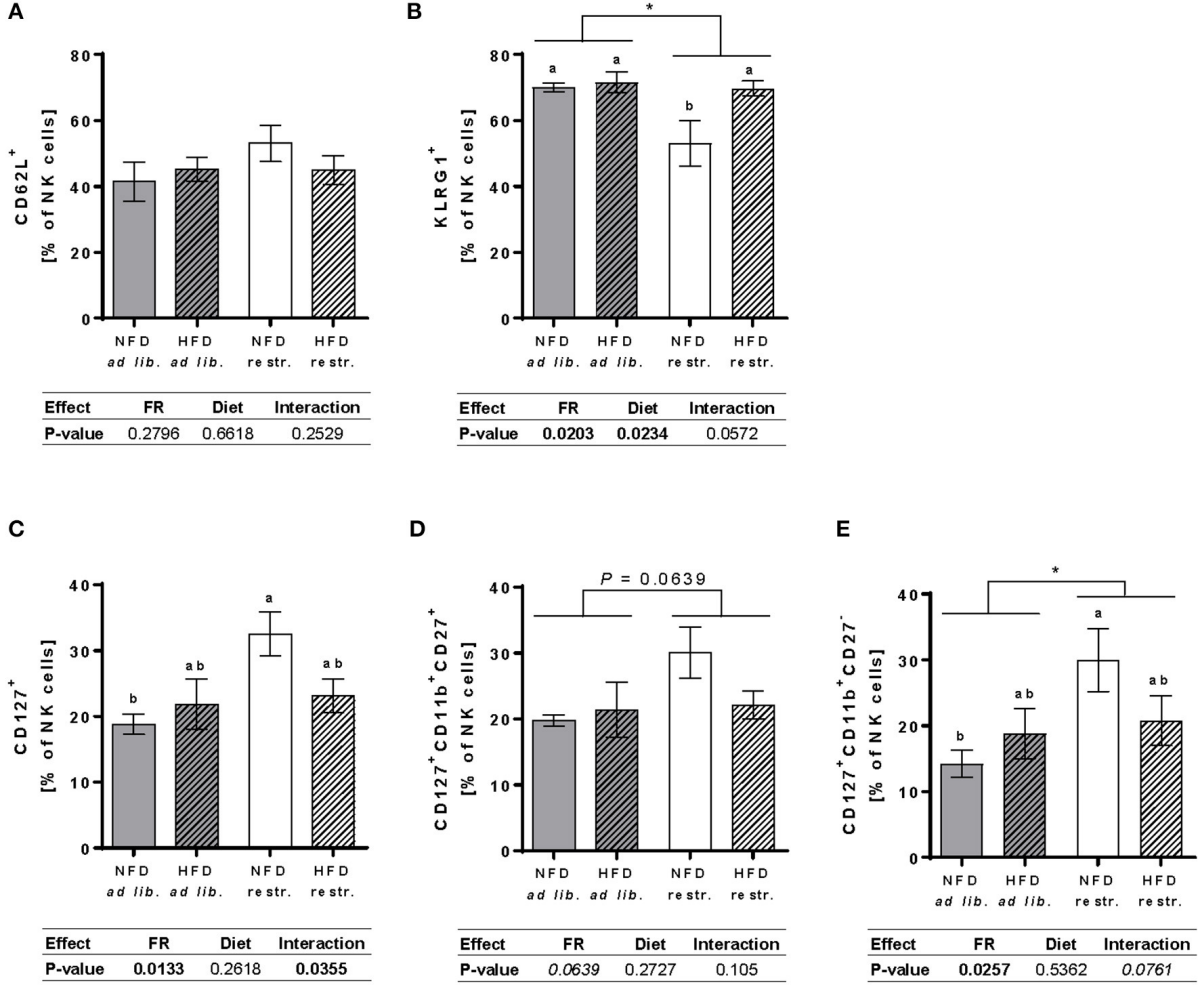
**(A,B)** Multicolor flow cytometric analyses of frequencies of total NK cells of BALB/c mice expressing the adhesion molecule CD62L **(A)** and the maturation marker killer cell lectin-like receptor G1 (KLRG1, B). **(C–E)** Frequencies of total NK cells, CD11b^+^CD27^+^NK cells and CD11b^+^CD27^−^ NK cells of BALB/c mice expressing the maturation marker CD127 **(B–D)**. Mice were fed a high-fat diet (HFD) or normal-fat diet (NFD) under *ad libitum* (ad lib.) or restrictive (restr.) feeding schemes. Data are presented as mean ± SEM. Different letters indicate means with significant differences according to *post-hoc* Tukey's multiple comparison test results (*P* ≤ 0.05). Data tables indicate *P*-values of two-way ANOVA analyses with the main factors “feeding regime (FR)” and “diet.” Significant differences are printed in bold type. Italic *P*-values indicate a tendency to significance (0.5 ≤ *P* ≤ 0.1). *P ≤ 0.05, two-way ANOVA, *ad libitum*-fed groups compared to restrictive-fed groups; exact *P*-values within 0.05 ≤ *P* ≤ 0.1 are depicted.

Investigations on the expression of the maturation marker KLRG1 on NK cells demonstrated a significantly reduced frequency of total NK cells expressing KLRG1 in restrictive-fed mice compared to *ad libitium*-fed mice unaffected by the diet. Moreover, feeding a HFD resulted in a significant increased frequency of total NK cells expressing KLRG1 compared to NFD-fed mice unaffected by the feeding regime. Analyses of single groups showed a significant reduction the frequency of total NK cells expressing KLRG1 in mice fed the NFD restrictively compared to the three other mice groups (Figure 6B). Analyses of the expression of the maturation marker CD127 in total NK cells and NK cell subsets revealed a significant increased frequency of total NK cells and the CD11b^+^CD27^−^ NK cell subset expressing CD127 in mice fed the NFD restrictively compared to mice fed the NFD *ad libitum* (Figures 6C,E). No significant differences were observed in the frequency of CD127^+^CD11b^+^CD27^+^ NK cells (Figure 6D). Moreover, expression analyses of the frequency of the adhesion molecule CD62L in total NK cells showed no significant differences between the four mice groups (Figure 6A).

### Real-Time RT-PCR Analyses of NK Cell Receptors and NK-Cell Related Parameters in Adipose Tissue

In addition to investigations on immune cell population and surface receptors on NK cells in peripheral blood, the relative mRNA expression of activating and inhibitory receptors, NKG2D ligands, NK cell-related transcription factors, and cytokines were analyzed in adipose tissue of the experimental animals (Table 1).

**Table 1:** Impact of high-fat diet (HFD) and normal-fat diet (NFD) on mRNA expression of NK cells, activating and inhibitory NK cell receptors, NK cell-related transcription factors, NKG2D-receptor ligands and cytokines under *ad libitum* and restrictive feeding regimes in visceral adipose tissue of BALB/c mice.

**Parameter**	**Name and alternative names**	***Ad libitum*** **feeding (mean** **±** **SEM)**		**Restrictive feeding (mean** **±** **SEM)**		**Two-way ANOVA (*****P*****-value)**		
		**NFD**	**HFD**	**NFD**	**HFD**	**Diet**	**Feeding regime**	**Diet x Feeding regime interaction**
Activating NK cell receptors	Fcgr3 (CD16)	1 ± 0.185	1.333 ± 0.276	0.587 ± 0.235	1.397 ± 0.192	**0.0189**	0.4437	0.299
	Klra4 (Ly49d)	1 ± 0.235	0.880 ± 0.149	1.202 ± 0.318	1.280 ± 0.259	0.9333	0.2308	0.6886
	Klra12 (Ly49l)	1 ± 0.192	1.385 ± 0.456	0.408 ± 0.165	1.412 ± 0.578	*0.0932*	0.4809	0.4418
	Klra22 (Ly49s2)	1 ± 0.202	0.771 ± 0.124	0.471 ± 0.164	0.972 ± 0.237	0.5	0.4159	*0.0802*
	Klrb1c (NK1.1, CD161)	1 ± 0.231	0.786 ± 0.136	0.660 ± 0.171	0.971 ± 0.172	0.8001	0.6859	0.1806
	Klrk1 (NKG2D)	1 ± 0.201	0.729 ± 0.067	0.950 ± 0.252	1.766 ± 0.527	0.4298	0.1599	0.1233
	NCR1 (NKp46, CD335)	1 ± 0.277	0.859 ± 0.108	0.600 ± 0.122	0.856 ± 0.106	0.7584	0.2855	0.2922
Activation associated NK cell receptor	CD69	1 ± 0.310	0.835 ± 0.176	0.871 ± 0.238	0.770 ± 0.209	0.6006	0.7015	0.8993
Co-activating receptor	CD244 (2B4)	1 ± 0.409	0.750 ± 0.183	0.400 ± 0.058	0.916 ± 0.233	0.6665	0.4836	0.2237
Inhibitory NK cell receptors	Klra1 (Ly49a)	1 ± 0.230	0.953 ± 0.134	0.810 ± 0.277	1.056 ± 0.145	0.6415	0.8377	0.4942
	Klra2 (Ly49b)	1 ± 0.113	0.890 ± 0.159	0.702 ± 0.132	0.835 ± 0.185	0.9414	0.2668	0.442
	Klra3 (Ly49c)	1 ± 0.209	0.857 ± 0.132	0.597 ± 0.130	0.915 ± 0.217	0.6441	0.3659	0.2299
	Klra5 (Ly49e)	1 ± 0.258	1.229 ± 0.294	0.812 ± 0.224	1.910 ± 0.556	*0.0831*	0.5064	0.2462
	Klra6 (Ly49f)	1 ± 0.206	1.251 ± 0.296	0.745 ± 0.217	1.666 ± 0.538	0.1044	0.8186	0.3427
	Klra7 (Ly49g)	1 ± 0.156	1.044 ± 0.127	0.614 ± 0.157	1.226 ± 0.402	0.1874	0.6752	0.2513
	Klra9 (Ly49i)	1 ± 0.226	0.931 ± 0.126	0.620 ± 0.161	1.503 ± 0.519	0.2373	0.7769	0.1696
	Klra10 (Ly49j)	1 ± 0.197	0.823 ± 0.125	0.487 ± 0.098	0.977 ± 0.236	0.4022	0.3384	*0.0829*
	Klrc1 (NKG2A)	1 ± 0.194	0.659 ± 0.155	0.713 ± 0.188	1.046 ± 0.169	0.9834	0.7857	*0.0767*
	Klrd1 (CD94)	1 ± 0.172	0.903 ± 0.097	0.858 ± 0.134	1.276 ± 0.303	0.4268	0.5656	0.2072
Co-inhibitory NK cell receptor	PDCD1 (PD-1, CD279)	1 ± 0.266	0.919 ± 0.209	0.753 ± 0.228	0.643 ± 0.145	0.6756	0.2571	0.9507
Transcription factors	Eomes	1 ± 0.194	1.054 ± 0.134	0.764 ± 0.327	0.933 ± 0.308	0.6646	0.488	0.8226
	Tbx21 (T-bet)	1 ± 0.160	0.907 ± 0.104	0.678 ± 0.074	0.990 ± 0.154	0.4336	0.3946	0.1561
NKG2D receptor ligands	Ulbp1 (MULT-1)	1 ± 0.090^b^	1.506 ± 0.115^ab^	1.019 ± 0.201^b^	2.018 ± 0.351^a^	**0.0026**	0.2374	0.2722
	Rea1 (RAE-1)	1 ± 0.151	1.067 ± 0.207	1.344 ± 0.271	0.672 ± 0.049	0.1225	0.893	*0.0633*
Cytokines	TNF-α	1 ± 0.152	1.424 ± 0.155	0.948 ± 0.246	1.607 ± 0.355	**0.0337**	0.7858	0.6275
	TNFsf10 (TRAIL)	1 ± 0.085	0.977 ± 0.120	1.145 ± 0.161	1.335 ± 0.266	0.6511	0.1806	0.5652

*CD, cluster of differentiation; Eomes, eomesodermin; Fcgr-3, Fc gamma receptor-3; HFD, high-fat diet; Klr, killer cell lectin-like receptor; MULT-1, mouse UL16-binding protein-like transcript 1; NCR, natural cytotoxicity receptor; NFD, normal-fat diet; NKG, natural killer group; PD-1, programmed cell death receptor-1; PPAR, peroxisome proliferator-activated receptor; Ppia, peptidylprolyl isomerase A; Rae-1, retinoic acid early inducible-1 gene; SEM, standard error of the mean; T-bet, T-cell associated transcription factor; Tbx21, T-box transcription factor 21; TNF, tumor necrosis factor; TRAIL, tumor necrosis factor related apoptosis inducing ligand; Ulbp-1, UL16-binding protein−1. Different superscript letters (a, b) indicate means that are significantly different according to post-hoc Tukey's multiple comparison test results (P ≤ 0.05). For two-way ANOVA analyses, P-values are shown for the main factors diet, feeding regime and the interaction of both main factors. Significant differences are printed in bold type. Italic P-values indicate a tendency to significance (0.5 ≤ P ≤ 0.1)*.

Analyzes on activating NK cell receptors in adipose tissue revealed that the relative mRNA expression of Fcgr3 (CD16) was significantly increased in restrictive-fed mice in comparison to *ad libitum*-fed mice unaffected by the diet. In contrast, no significant changes between the four experimental groups were observed for the relative mRNA expression of the activating NK cell receptors Klra4 (Ly49d), Klra12 (Ly49l), Klra22 (Ly49s2), Klrb1c (NK1.1, CD161), Klrk1 (NKG2D) and NCR1 (NKp46, CD335) as well as the activation-associated receptor CD69 and the co-activating receptor CD244 (2B4).

Moreover, investigations on inhibitory NK cell receptors in adipose tissue demonstrated no significant differences in the relative mRNA expression of the inhibitory NK cell receptors KLra1 (Ly49a), Klra2 (Ly49b), Klra3 (Ly49c), Klra5 (Ly49e), Klra6 (ly49f), Klra7 (Ly49g), Klra9 (Ly49i), Klra10 (Ly49j), Klrc1 (NKG2A) and Klrd1 (CD94) as well as the co-inhibitory NK cell receptor PDCD1 (PD-1, CD279) comparing the four experimental groups. In addition, the relative mRNA expression of the transcription factors Eomes and Tbx21 (T-bet) in adipose tissue of BALB/c mice was not affected by feeding a high-fat diet or restrictive feeding.

Interestingly, results demonstrated a significant increase in the relative mRNA expression levels of the NKG2D receptor ligand Ulbp1 (MULT-1) in mice fed the HFD in comparison to mice fed the NFD unaffected the feeding scheme. Single-group analyses demonstrate a significant increase of the relative Ulbp1 (MULT-1) mRNA expression in mice fed the HFD restrictively compared to mice fed the NFD *ad libitum* and mice fed the NFD restrictively. Restrictive feeding or feeding a HFD did not affect on the relative mRNA expression of the NKG2D receptor ligand Rae1 (RAE-1).

In adipose tissue, results also revealed a significant increase in the relative mRNA expression levels of the pro-inflammatory cytokine TNF-α of mice fed the HFD in comparison to mice fed the NFD unaffected by the feeding regime. In contrast, no significant effects of the diet or feeding regime on the relative mRNA expression levels of the cytokine TNFsf10 (TRAIL) were detected.

## Discussions

Numerous animal studies revealed obesity-related alterations in number, phenotype, cytokine secretion as well as cytotoxicity of NK cells in different diet-induced obese rat strains and diet- or genetically-induced obese C57BL/6 mice (10, 18). However, in these studies an *ad libitum*-feeding regime was used, and the obtained data revealed no information whether the effects on NK cells are a consequence of obesity or induced by the dietary composition and the high fat content *per se*. In contrast to previous studies, the present study aimed to investigate the impact of high-fat feeding on NK cells in obese-resistant female BALB/c mice which either were fed a normal fat or high-fat diet in an *ad libitum* or restrictive feeding scheme.

In line with previous studies, results demonstrate that *ad libitum-*fed BALB/c mice were resistant to increase body weights and fat mass after feeding a HFD compared to mice fed the NFD *ad libitum* (27, 28). Interestingly, feeding a HFD in a restrictive manner resulted in significantly higher body weight and visceral fat mass compared to mice fed the NFD restrictively, indicating that BALB/c mice fed the HFD loose lower weight compared to NFD mice fed in a restrictive manner. These data are very important as they shed light into the importance of feeding regimes, heavily discussed in these days. Moreover, analyses of plasma cytokines demonstrated that restrictive feeding leads to a significant decrease of the pro-inflammatory plasma cytokine IFN-γ, especially in NFD-fed mice. In line with previous results, *ad libitum*-feeding of a HFD in BALB/c mice had no effects on pro-inflammatory cytokines (29).

Own and other previous studies using an *ad libitum* feeding regime reported an increase in lymphocyte and granulocyte cell numbers in the bone marrow and blood of HFD-fed obese C57BL/6 mice in comparison to control mice (18, 30). In contrast, results of the present study did not find any effects of HFD-feeding in total lymphocyte counts or the frequency of lymphocytes and granulocytes in *ad libitum*-fed BALB/c mice, but data demonstrate that restrictive HFD-feeding led to significantly increased total lymphocyte counts as well as a decreased frequency of granulocytes compared to restrictive feeding of a NFD. These data indicate that the HFD-induced changes in plasma cytokine levels as well as in the frequencies of lymphocytes and granulocytes are rather an effect of the obese metabolic state than a direct diet-induced effect. In line with other studies, but in contrast with our study on C57BL/6 mice, the present analyses of immune cell population also revealed significantly enhanced frequencies of CD8^+^ T cells in HFD-fed mice in comparison to NFD-fed mice unaffected by the feeding regime (18, 31). As CD8^+^ T cells have been, described to be involved in promoting inflammatory responses in obese adipose tissue, the increase of CD8^+^ T cells in HFD-fed mice may be related in the pathomechanism of the obesity-associated inflammation (32).

Previous investigations in rodents commonly measured either the frequency or the total number of NK cells, providing conflicting results about obesity-associated effects of blood NK cells (10). As the proportion of other leucocytes may influence the frequency of NK cells, we determined both parameters, the total cell count as well as the frequency of NK cells. A significant increase of total NK cell number was shown in blood of mice fed the HFD compared to mice fed the NFD. Interestingly, neither the feeding regime nor the diet had significant effects on the frequency of peripheral blood NK cells in BALB/c mice, whereas the number of peripheral blood NK cells was significantly reduced in restricted-fed mice compared to mice fed *ad libitum*-fed BALB/c mice. In contrast, own previous studies on C57BL/6 mice showed no significant effects of restricted feeding on NK cell number or frequency (18).

Detailed analyses of the four murine NK cell subpopulations in BALB/c mice demonstrated that restricted feeding results in an increase of the more immature CD11b^−^CD27^+^ and CD11b^+^CD27^+^ NK cell subsets and a decrease of the most mature CD11b^+^CD27^−^ NK cell subset. In contrast, recent own experiments on C57BL/6 mice revealed no significant effects of restricted feeding on NK cell subpopulations (18).

Investigating the impact of HFD feeding, results of the present study revealed that feeding a HFD leads to a decreased frequency of CD11b^−^CD27^+^ NK cells and a slightly, but not significantly, increased frequency CD11b^+^CD27^−^ NK cells in peripheral blood of restrictively-fed BALB/c mice. However, no HFD-induced changes in NK cell subsets were observed in *ad libitum*-fed BALB/c mice, which is probably contributed to the obese-resistance in the *ad libitum*-fed mice. These data are in line with previous studies on diet-induced obese C57BL/6 mice demonstrating a decrease of the CD11b^−^CD27^+^ NK cell subset and an increase of CD11b^+^CD27^−^ NK cells in visceral adipose tissue, but no HFD diet-induced changes on NK cell subpopulations in blood (33, 34). In contrast, our study on C57BL/6 mice revealed significantly decreased frequencies of the CD11b^+^CD27^+^ NK cell subset with a marked difference comparing HFD *ad libitum*- and NFD restrictive-fed mice (18).

These discrepancies of the results indicate that there are strain and diet-specific differences on the effects of restricted feeding and feeding a HFD on frequency, number and subpopulations of NK cells in mice.

In addition to differences in total NK cell number and NK cell subpopulations, results of the present study revealed, for the first time, that restrictive feeding leads to an impaired NK cell phenotype in peripheral blood NK cells of BALB/c mice. Data demonstrate a significantly reduced frequency of NK cells expressing the KLRG1, a maturation marker, as well as an increased frequency of the maturation marker CD127 in total NK cells of restrictive-fed BALB/c mice. Interestingly, the present results of a decreased mature NK cell subset associated with enhanced frequencies of CD127^+^ NK cells and decreased frequency of KLRG1^+^ NK cells confirm previous results detected in spleens of restrictively-fed C57BL/6 mice (24). Present analyses of CD127 expression on NK cell subsets also demonstrate that restricted feeding predominantly increased the frequency of CD127^+^CD11b^+^CD27^−^ NK cells and only slightly, but not significantly, increased the frequency of CD127^+^CD11b^+^CD27^+^ NK cells. As CD127 was described a marker for immature NK cells, these data indicate that a moderate dietary restriction negatively influences maturation and differentiation of murine NK cells.

In addition to effects of the feeding regime, we also detected alterations in the expression of the KLRG1, a maturation marker, in mice fed the HFD restrictively in comparison to mice fed the NFD restrictively, whereas no HFD-induced effects were found in *ad libitum*-fed mice. These data indicate that not only dietary restriction, but also feeding a HFD modulates NK cell maturation and differentiation. As KLRG1 and CD127 are known to play a role in the regulation of NK cell cytotoxicity, the alterations of the expression of these maturation markers induced by feeding high-fat diet or restrictive feeding may also affect the NK cell killing capacity (35–37).

In addition to results of NK cell maturation markers, data of the present study demonstrate an enhanced expression of the activation-associated receptor CD69 in restrictive-fed mice in comparison to *ad libitum*-fed mice independent of the diet. As the CD69 receptor is involved in promoting NK cell cytotoxicity, these data indicate that restrictive feeding leads to an increased cytolytic capacity of NK cells in BALB/c mice (38). In contrast, data of the present study demonstrate a decreased expression of the NKG2D, an activating receptor, in restrictive-fed mice. Previous studies demonstrate that engagement of NKG2D can trigger cytolytic activity as well as cytokine production (39). Therefore, a decreased NKG2D receptor expression on NK cells of restrictive-fed mice may lead to a reduced NK cell killing capacity. Future studies investigating the impact of restricted feeding on cytokine secretion and cytotoxicity are mandatory to interpret the effects of a restricted dietary intake on NK cell functionality.

Although some data on altered gene expression profiles of NK cell receptors in peripheral blood or tissues in mice and rats investigating the influence of diet-induced obesity are published, only own data on the protein surface expression of NK cell receptors on blood NK cells in C57BL/6 mice exist until now (10). These data revealed enhanced expression of maturation markers on NK cells in HFD-fed mice. Interestingly, present data demonstrate that *ad libitum*-feeding of a HFD did not influence the distribution of NK cell subpopulation as well as the surface expression of NK cell receptors in obese-resistant BALB/c mice. In contrast, restrictive-feeding of a HFD to BALB/c mice, led to a significantly increased body weight and visceral fat mass which was associated with changes in the frequencies of NK cell subsets and NK cell phenotype.

As only a rare studies investigated the influence of obesity on NK cell-related functional marker in adipose tissue, we also analyzed the relative mRNA expression of activating and inhibitory NK cell receptors, NKG2D ligands, NK cell-related transcription factors, and cytokines. The increased relative mRNA expression of the pro-inflammatory cytokine TNF-α in adipose tissue of HFD-fed mice once more confirms the increased adipose tissue inflammation in obesity. Results also demonstrate an increased relative mRNA expression of the activating NK cell receptor Fcgr3 (CD16) in mice fed the HFD, which is in line with the results of our study on C57BL/6 mice (18). Accordingly, previous studies in diet-induced obese C57BL/6 mice as well as in obese humans demonstrated an increased frequency of NK cells expressing activating NK cell receptors in adipose tissue (34, 40). Furthermore, results demonstrate an upregulation of the relative mRNA expression of the NKG2D receptor ligand Ulbp1 (MULT-1) in HFD-fed compared to NFD-fed BALB/c mice. These data are in line with previous findings of an upregulation of NKp46 and NKG2D ligands in adipose tissue of HFD-fed C57BL/6 mice and confirm the results of our previous studies on C57BL6 mice and BALB/c mice (18, 33, 41, 42). In sum, these data indicate that HFD-feeding may induce a more active status of NK cells in adipose tissue. As NK cells have been shown to trigger the differentiation of the anti-inflammatory M2 macrophages to the pro-inflammatory M1 macrophages, they have been identified to be involved in inflammatory processes the adipose tissue (10, 33).

In conclusion results of the present study robustly reveal that even a moderate restriction of the availability and amount of the diet can partially attenuate the obesity-associated alterations of NK cells in mice. These data can help to clarify the increased risk for cancer and severe infections, like infections with SARS-CoV-2, in obesity. Moreover, in contrast to obese-prone C57BL/6 mice, *ad libitum* feeding of a HFD in obese-resistant BALB/c mice had no effects on body weight gain, visceral fat mass as well as the number, frequency and phenotype of NK cells. Therefore, we conclude that previously observed HFD-induced alterations in NK cells are consequences of the obese associated immunological profile rather than a direct effect of the dietary composition. Future investigations on obesity-prone mouse strains and humans are necessary to investigate the impact of dietary restriction in diet-induced obesity on NK cells. These data encourage everyone to mind a thoughtful lifestyle and to consider interventions once an excess of body weight has occurred.

## Data Availability Statement

The original contributions presented in the study are included in the article/Supplementary Material, further inquiries can be directed to the corresponding author/s.

## Ethics Statement

The animal study was reviewed and approved by State administration of Saxony-Anhalt, Animal welfare committee, Dessauer Strasse 70, 06118 Halle (Saale), Germany.

## Author Contributions

JS and IB planned, conducted, and supervised the experimental study, supported by HK. WN, MA, and ME executed the animal experiments under supervision of IB and JS. The design of the flow cytometric set up and analyses workflow as well as was the flow cytometry analyses were conducted by DQ. JS and IB were main contributors in authoring the manuscript with support from WN. DQ, JF, and HK revised the manuscript critically. All authors read and approved the final manuscript.

## Conflict of Interest

The authors declare that the research was conducted in the absence of any commercial or financial relationships that could be construed as a potential conflict of interest.

## Publisher's Note

All claims expressed in this article are solely those of the authors and do not necessarily represent those of their affiliated organizations, or those of the publisher, the editors and the reviewers. Any product that may be evaluated in this article, or claim that may be made by its manufacturer, is not guaranteed or endorsed by the publisher.
