# Supplementary material for: The Impact of High-Fat Diet and Restrictive Feeding on Natural Killer Cells in Obese-Resistant BALB/c Mice
Source: Front Nutr. 2021 Jul 23;8:711824. doi: 10.3389/fnut.2021.711824 (PMC8342926; doi:10.3389/fnut.2021.711824)
Supplement: Supplementary file 1 [file Presentation_1.pdf]

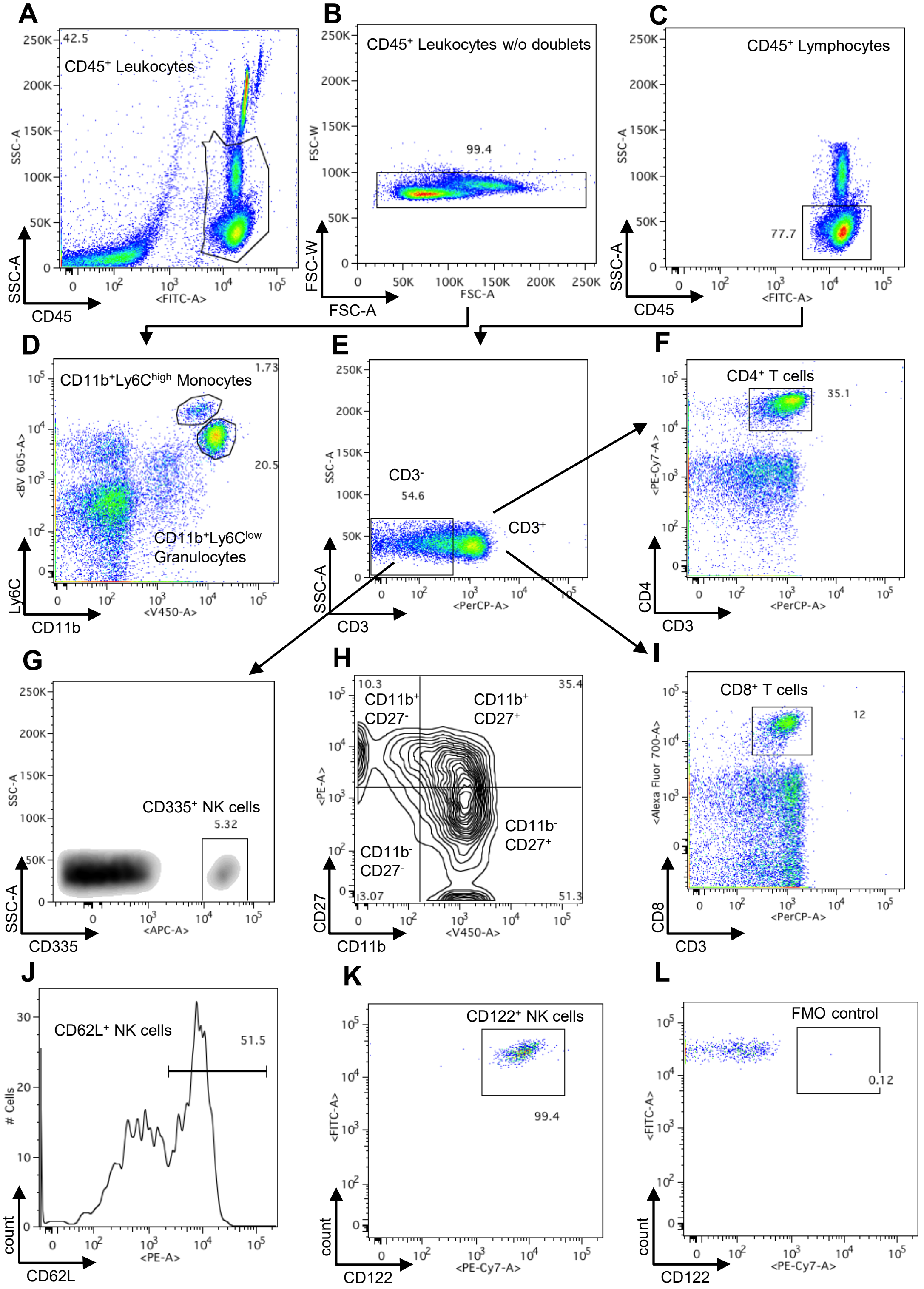

Supplementary figure 1: Representative plots of the hierarchical gating strategy to identify immune cell populations, NK cell subsets and NK cell surface marker expression from BALB/c mice. Leucocytes were identified by their CD45 expression and granularity using SSC (A), followed by doublet exclusion (B), and gating of CD45^{+} SSC^{low} lymphocytes (C). Leucocyte gating combined with single cell gating leads to the identification of CD11b^{+}Ly6C^{high} monocytes and CD11b^{+}Ly6C^{low} granulocytes (D). Based on the lymphocyte gate, CD3^{-} and CD3^{+} lymphocytes (E) were classified. CD3^{+}CD4^{+} helper T cells (F) and CD3^{+}CD8^{+} cytotoxic T cells (I) were identified from the CD3^{+} fraction. CD335^{+} NK cells were identified from the CD3^{-} fraction (G). On the basis of the expression of CD11b and CD27, the total NK cell population was further differentiated into four NK cell subsets (H). The expression of different NK cell marker were assessed on the CD335^{+}-gated NK cell fraction. Representative images of CD62L as histogram (J) and of CD122 using dot plot are given (K). FMO controls were used to determine positive cells, exemplified for CD122 staining (L).
