# Supplementary material for: The Impact of High-Fat Diet and Restrictive Feeding on Natural Killer Cells in Obese-Resistant BALB/c Mice
Source: Front Nutr. 2021 Jul 23;8:711824. doi: 10.3389/fnut.2021.711824 (PMC8342926; doi:10.3389/fnut.2021.711824)
Supplement: Supplementary file 2 [file Table_1.pdf]

Supplementary table 1: Composition of experimental control and high-fat diet.

Nutrients	Control diet (D12450J)	High-fat diet (12492)
Energy (kcal/g)	3.85	5.24
Protein (g/kg)	192 (20 kcal%)	262 (20 kcal%)
Carbohydrate (g/kg)	673 (70 kcal%)	263 (20 kcal%)
Fat (g/kg)	43 (10 kcal%)	349 (60 kcal%)
Casein (g/kg)	189.6	258.4
L-Cysteine (g/kg)	2.8	3.9
Corn starch (g/kg)	479.8	0
Maltodextrin (g/kg)	118.5	161.5
Sucrose (g/kg)	65.2	88.9
Cellulose (g/kg)	47.4	64.6
Sybean oil (g/kg)	23.7	32.3
Lard (g/kg)	19.0	316.6
Mineral mix (g/kg)	9.5	12.9
Cholesterol (mg/kg)	51.6	300.8
Dicalcium phosphate (g/kg)	12.3	16.8
Calcium carbonate (g/kg)	5.2	7.1
Potassium citrate (g/kg)	15.6	21.3
Vitamin mix (g/kg)	9.5	12.9
Choline bitartrate (g/kg)	1.9	2.6
