# Supplementary material for: The Impact of High-Fat Diet and Restrictive Feeding on Natural Killer Cells in Obese-Resistant BALB/c Mice
Source: Front Nutr. 2021 Jul 23;8:711824. doi: 10.3389/fnut.2021.711824 (PMC8342926; doi:10.3389/fnut.2021.711824)
Supplement: Supplementary file 3 [file Table_2.pdf]

Supplementary table 2: Fluorochrome-conjugated mononuclear antibodies for surface staining of murine peripheral blood immune cells for flow cytometric analysis.

Antigen	Fluorochrome	Isotype	Clone	Concentration [µg/ml]	Company
CD3e	PerCP	Hamster IgG1, κ	145-2C11	100.00	BD Biosciences
Ly-6G	BV510	Rat IgG2a, κ	1A8	50.00	BD Biosciences
Ly-6C	BV605	Rat IgM, κ	AL-21	200	BD Biosciences
KLRG1	BV421	Hamster IgG2, κ	2F1	50.00	BD Biosciences
CD8a	Alexa Fluor 700	Rat IgG2a, κ	53-6.7	100.00	BD Biosciences
CD4	PE-Cy	Rat IgG2a, κ	RM4-5	100.00	BD Biosciences
CD45	FITC	Rat IgG2b, κ	30F11	150.00	Miltenyi Biotec
CD27	PE	Hamster IgG	LG.3A10	30.00	Miltenyi Biotec
CD127	PE-Vio770	Rat IgG2aκ	A7R 34	30.00	Miltenyi Biotec
CD335 (NKp46)	APC	Rat IgG2aκ	29A1.4.9	150.00	Miltenyi Biotec
CD122 (IL-2Rβ)	PE-Vio770	Rat IgG2bκ	TM-β1	30.00	Miltenyi Biotec
CD94	APC-Vio770	Rat IgG2aκ	18d3	30.00	Miltenyi Biotec
CD69	PE	Hamster IgG1	H1.2F3	30.00	Miltenyi Biotec
CD314 (NKG2D)	PE-Vio770	Rat IgG1κ	CX5	30.00	Miltenyi Biotec
CD62L	PE	Rat IgG2aκ	MEL14-H2.100	30.00	Miltenyi Biotec
Ly-49C/F/I/H	APC-Vio770	Hamster IgG	14B11	30.00	Miltenyi Biotec
CD244.1 (2B4)	PE-Vio770	Recombinant human IgG1	REA388	30.00	Miltenyi Biotec
CD19	APC-Vio770	Rat IgG2aκ	6D5	30.00	Miltenyi Biotec
CD11b	VioBlue	Recombinant human IgG1	M1/70.15.11.5	33.00	Miltenyi Biotec
CD49b	PE	Rat IgM κ	DX5	30.00	Miltenyi Biotec

APC, allophycocyanin; BV, brilliant violet; CD, cluster of differentiation; Cy, cyanine; FITC, fluorescein isothiocyanate; Ig, immunoglobulin; IL-2R, interleukin-2 receptor; Klr, killer cell lectin-like; NKG2, natural killer group two; PE, phycoerythrin; PerCP, peridinin chlorophyll protein;
BD Biosciences, San Jose, CA, USA; Miltenyi Biotec, Bergisch Gladbach, Germany.
