# Supplementary material for: The Impact of High-Fat Diet and Restrictive Feeding on Natural Killer Cells in Obese-Resistant BALB/c Mice
Source: Front Nutr. 2021 Jul 23;8:711824. doi: 10.3389/fnut.2021.711824 (PMC8342926; doi:10.3389/fnut.2021.711824)
Supplement: Supplementary file 4 [file Table_3.pdf]

Supplementary table 3: Parameters of primers used for real-time RT-PCR analyses.

**Gene**	**Primer sequence (5`-3`)**		**Product size (bp)**	**NCBI gene bank reference**
	**Forward**	**Reverse**		
2B4 (CD244)	CAGTATATTCAGTAGTCCAGC	CACGGTACAACTTAAGGAAG	80	NM_018729
CD69	AAAAGGACATGACGTTTCTG	CAGCTGTTAAATTCTTTGCC	115	NM_001033122
Eomes	ACAACACACAGATGATAGTG	TATGGTCGATCTTTAGCTGG	195	NM_001164789
Fcgr3 (CD16)	ATTTCTCTATCCCAAAAGCC	CTAGAGAGATGGAGGATGTAG	138	NM_010188
Klra1 (Ly49a)	ATGGACAGAAAAACATGGAG	TTCAAGGCAATTTAGATGG	200	NM_016659
Klra2 (Ly49b)	CAAGGGAAGAACTCAATCTG	GACACAAGCTTCTCTGTTTAC	120	NM_001170851
Klra3 (Ly49c)	CAGAGGTGTTAAATACTGGTTC	ATTCTCTGGAATAACATGGC	172	NM_010648
Klra4 (Ly49d)	TTTCGTCATGGACAGAAAAC	GAACTTCAGTTCATCCTCATC	100	NM_010649
Klra5 (Ly49e)	ACAAGAAATCCACGAAACTC	CTTGGTTTCACTGTACCATC	169	NM_008463
Klra6 (Ly49f)	TGACAATGGCCAATCTAAAC	TCTCTATTCACAGCAGTCTATG	200	NM_008464
Klra7 (Ly49g)	AAGCTCATTGTGATAGCTTG	GCTGAAAAATCGTTATTGCC	82	NM_001110323
Klra9 (Ly49i)	CGTTCCCATTGTGAAGATAG	GACAATCCAATCCAGTAACTC	87	NM_010651
Klra10 (Ly49j)	CCCATTGTGAAGATAGAAGATG	CATGTCAAGTTTAGATGGGC	144	NM_008459
Klra12 (Ly49l)	ACTGAAGTTCCTTAAGCTCC	ACAACATACATCCTCCATCTC	158	NM_010646
Klra22 (Ly49s2)	GATAGACAATGAGGATGAACTG	GTTCAAGGCAAGCTTAGATG	136	NM_053152
Klrb1c (NK1.1, CD161)	AACTGAGATTCCTACTGGAC	TTGTGCCATTTATCCACTTC	105	NM_001159904
Klrk1 (NKG2D)	AGTATTGTGCAACAAGGAAG	TTTTGAGACAACCAGGAAGC	153	NM_033078.4
Klrc1 (NKG2A)	CCCACAGAGATATAAACTACAC	TGCTCCTCTTCACTATCTATG	184	NM_001136068
Klrd1 (CD94)	AAGTCTTGGAAAAGAAGCAG	GCATTCCAATCCAGAAAAAG	121	NM_010654
NCR1(CD335, NKp46)	TAGTAACTGGTCTGTATGACAC	CTTGAGCAGAAAGAATTTGC	125	NM_010746
PDCD1 (PD-1)	ACTAGGGCAATAAAGGGAAC	GAATGAGGAGATTCTAACACC	176	NM_008798
PPAR-α	GATGTCACACAATGCAATTC	CAGTTTCCGAATCTTTCAGG	107	NM_001113418
Ppia	CACCGTGTTCTTCGACATCA	TGTCTGCAAACAGCTCGAAG	71	NM_008907.1
Rae-1(RAE-1)	CAATGAAGGATATCGAAGTGAC	GAAGTTGCCTGGTAAAGTTG	86	NM_175112
Tbx21 (T-bet)	ACGTCTTTACTTTCCAAGAG	GTACATGGACTCAAAGTTCTC	128	NM_019507
TNF-α	CTATGTCTCAGCCTCTTCTC	CATTTGGGAACTTCTCATCC	125	NM_013693
TNFsf10 (TRAIL, CD253)	GAAAAGCAGCTAAGTACTCC	ACGTGGTTGAGGAAATGAATG	189	NM_009425.2
Ulbp-1 (MULT-1)	ACCTGTGTTTATGCAGATTG	CCCATCAATATCGTCTGAAG	154	NM_029975

bp, base pair; CD, cluster of differentiation; EOMES, eomesodermin; Fcgr-3; Fc gamma receptor-3; Klr, killer cell lectin-like receptor; MULT-1, mouse UL16-binding protein-like transcript 1; NCBI, National Center for Biotechnology Information; NCR, natural cytotoxicity receptor; NKG, natural killer group; PD-1; programmed cell death receptor-1; PPAR, peroxisome proliferator-activated receptor; Ppia, peptidylprolyl isomerase A; Rae-1, retinoic acid early inducible-1 gene; T-bet, T-cell associated transcription factor; Tbx21, T-box transcription factor 21; TNF, tumor necrosis factor; TRAIL, tumor necrosis factor related apoptosis inducing ligand; Ulbp-1, UL16-binding protein -1.
