# Supplementary material for: The Impact of High-Fat Diet and Restrictive Feeding on Natural Killer Cells in Obese-Resistant BALB/c Mice
Source: Front Nutr. 2021 Jul 23;8:711824. doi: 10.3389/fnut.2021.711824 (PMC8342926; doi:10.3389/fnut.2021.711824)
Supplement: Supplementary file 5 [file Table_4.pdf]

Supplementary table 4: Nutritional data of calculated daily dietary intake in BALB/c mice fed a normal-fat diet (NFD) or high-fat diet (HFD) with *ad libitum* or restrictive feeding regimes.

	Ad libitum feeding (Mean ± SEM)		Restrictive feeding (Mean ± SEM)		Two-way ANOVA (P-value)		
Nutritional component	NFD	HFD	NFD	HFD	Diet	Feeding regime	Diet x Feeding regime interaction
Food amount [g/day]	3.012 ± 0.025[a]	2.141 ± 0.046[c]	2.706 ± 0.013[b]	1.940 ± 0.006[d]	**< 0.0001**	**< 0.0001**	**0.0480**
Energy [kcal/day]	11.60 ± 0.095[a]	11.22 ± 0.240[a]	10.42 ± 0.051[b]	10.17 ± 0.032[b]	**0.0161**	**< 0.0001**	0.6081
Fat [g/day]	0.130 ± 0.00[c]	0.747 ± 0.016[a]	0.116 ± 0.001[c]	0.677 ± 0.002[b]	**< 0.0001**	**< 0.0001**	**0.0006**
Protein [g/day]	0.578 ± 0.005[a]	0.561 ± 0.010[a]	0.520 ± 0.003[b]	0.508 ± 0.002[b]	**0.0269**	**< 0.0001**	0.6165
Carbohydrate [g/day]	2.027 ± 0.017[a]	0.563 ± 0.012[c]	1.821 ± 0.009[b]	0.510 ± 0.002[d]	**< 0.0001**	**< 0.0001**	**< 0.0001**

HFD, high-fat diet; NFD, normal-fat diet; SEM, standard error of the mean. Different superscript letters (a,b,c,d) indicate significant differences between individual experimental groups analyzed by Tukey´s multiple comparison test ($p \leq 0.05$). For two-way ANOVA analyses, p-values are shown for the main factors diet, feeding regime and the interaction of both main factors. Significant differences are printed in bold type.
